# The attitudinal space framework: Embracing the multidimensionality of attitudinal diversity

**DOI:** 10.1016/j.isci.2023.107340

**Published:** 2023-07-16

**Authors:** Ugo Arbieu, Jörg Albrecht, Katrin Böhning-Gaese, Lisa Lehnen, Matthias Schleuning, Thomas Mueller

**Affiliations:** 1 Laboratoire d’Ecologie Systématique et Evolution, IDEEV, Université Paris-Saclay, 91190 Gif-sur-Yvette, France; 2 Senckenberg Biodiversity and Climate Research Centre (SBiK-F), Senckenberganlage, 60325 Frankfurt am Main, Germany; 3 Smithsonian Conservation Biology Institute, National Zoological Park, 1500 Remount Road, Front Royal, VA 22630, USA; 4 Department of Biological Sciences, Goethe University Frankfurt am Main, 60438 Frankfurt am Main, Germany

**Keywords:** Nature conservation, Ecology, Social sciences, Psychology

## Abstract

Attitude polarization describes an increasing attitude difference between groups and is increasingly recognized as a multidimensional phenomenon. However, a unified framework to study polarization across multiple dimensions is lacking. We introduce the attitudinal space framework (ASF) to fully quantify attitudinal diversity. We highlight two key measures—attitudinal extremization and attitudinal dispersion—to quantify across- and within-group attitudinal patterns. First, we show that affective polarization in the US electorate is weaker than previously thought based on mean differences alone: in both Democrat and Republican partisans, attitudinal dispersion increased between 1988 and 2008. Second, we examined attitudes toward wolves in Germany. Despite attitude differences between regions with and without wolves, we did not find differences in attitudinal extremization or dispersion, suggesting only weak attitude polarization. These results illustrate how the ASF is applicable to a wide range of social systems and offers an important avenue to understanding societal transformations.

## Highlights

• Attitude polarization is increasingly understood as a multidimensional phenomenon
• We introduce the attitudinal space framework to quantify attitudinal diversity
• Affective polarization in the US electorate may be weaker than previously thought
• In a second case study we found weak polarization in attitudes toward wolves in Germany

## Graphical abstract

**Figure:**
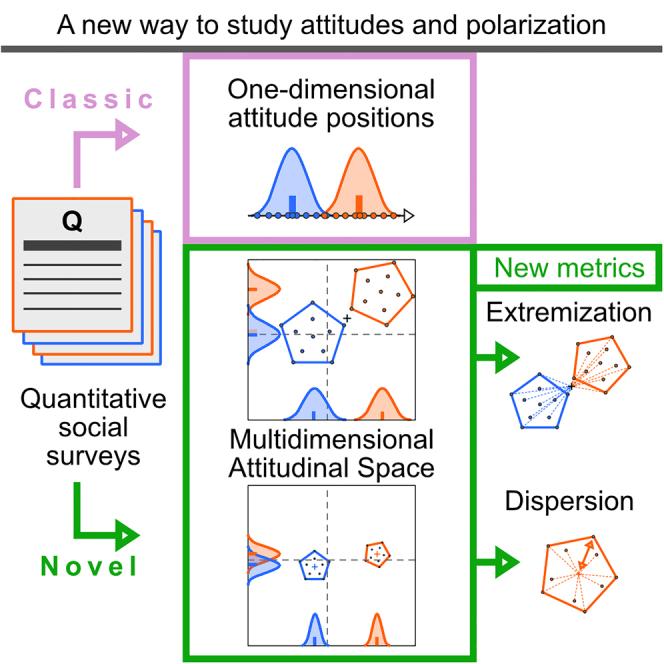

## Introduction

### Attitude polarization: A societal issue of high relevance

Attitudes are an important component of human cognition as they represent a critical link between societal norms and people’s behavior.^1^ Understanding individual attitudes and their dynamics is therefore central to address political,^2^ societal,^3^ economic,^4^ technological,^5^ or environmental issues.^6^ The phenomenon of attitude polarization is rooted in social identity theory^7^ and is generally described as either the difference (when understood as a state) or the increasing difference (when understood as a trend) in attitudes between two social groups.^8^^,^^9^ For instance, ideological polarization reflects the increasing divide in public opinion over several societal issues,^10^^,^^11^^,^^12^ and affective polarization depicts the increasing animus between opposite partisan groups.^13^^,^^14^ The concept of polarization has recently gained attention as it has wide-ranging implications in society, from affecting voting behavior to generating conflicts over wildlife management or hindering transformative change toward sustainability, and is therefore relevant to various fields of research. We here make two important distinctions. First, the focus of this article is on attitude polarization, in contrast to the broader concept of polarization in society, which often manifests in attitude polarization but is not limited to it. For instance, polarization in society can be observed through multiple processes like biased communication and disinformation,^15^ political elites’ behavior, discrimination, trust issues, and weakened social connections,^16^^,^^17^ to the extent of political sectarianism.^18^ Second, there exists an important difference between actual and perceived polarization.^19^^,^^20^^,^^21^ While actual polarization (like ideological or affective polarization) objectively reflects how individuals position themselves along societal issues, perceived polarization reflects people’s first-order (what I think *they* believe about the issue) and second-order judgments (what I think *they* believe about us).^19^^,^^22^ Recent work on actual vs. perceived polarization has demonstrated that the two concepts are interlinked^23^ and that perceived polarization is, most often than not, inaccurate and exaggerated (“false polarization”),^24^ hence reinforcing actual polarization.^19^^,^^25^ In this article, we focus on actual attitude polarization (“attitude polarization” henceforth), to highlight limitations in previous study and introduce a complementary framework to existing ones to better understand the complexity of attitude polarization.

Attitude polarization, across disciplines, has been relatively limited to the study of attitude positions, i.e., mean group attitudes.^8^^,^^26^^,^^27^^,^^28^ These approaches are insufficient to unravel the mechanisms behind the two main aspects of polarization: the increase in across-group distances and the reduction of within-group dispersion,^29^^,^^30^ which have been studied separately so far. We therefore currently lack a unified quantitative framework to study these aspects together. In this article, we present the attitudinal space framework (ASF), a novel, unifying framework that allows to jointly evaluate within- and across-group attitudinal dynamics across multiple attitude dimensions.

### The need for multidimensional assessments of polarization

The recognition that attitude polarization is a multidimensional phenomenon has recently gained momentum.^10^^,^^31^^,^^32^^,^^33^ In particular, multidimensional approaches offer the possibility to address fundamental aspects of polarization like issue alignment and group homogeneity. Issue alignment occurs when attitudes of individuals belonging to the same social group tend to converge on multiple issues.^9^^,^^32^^,^^34^ These multiple issues, or attitudinal dimensions, have traditionally been investigated separately; however, multidimensional assessments can reveal whether polarization over one attitudinal dimension entails polarization also on other dimensions.^35^^,^^36^ Group homogeneity represents the extent to which social groups are clearly defined with sharp boundaries.^31^^,^^37^ It is subject to competing hypotheses in the context of attitude polarization: some argue that social groups are expected to become internally more homogeneous as they polarize.^37^ For instance, in the political realm, proponents of the hypothesis of party sorting claim that partisans, through the influence of interactions with like-minded family and social networks, are expected to become more politically homogeneous.^38^ This would result in a strong attitude polarization where both attitude differences and group homogeneity increase and contribute to polarization. In contrast, others argue that polarization need not mean greater homogeneity within social groups.^39^ For instance, proponents of the dual motivation hypothesis suggest that polarization leaves ample room for discord within political parties. The multiplicity of independent individual thinking within partisan groups can reduce homogeneity in partisans’ attitudes,^14^ thus resulting in an overall weaker polarization phenomenon.

Issue alignment and group homogeneity are key to understand across- and within-group dynamics because, together, they can help differentiate between weak and strong polarization. Strong polarization can be characterized by an increasing distance between two social groups’ attitude positions, in combination with high issue alignment (convergence of several attitude positions) and within-group homogeneity. On the opposite, absence of strong issue alignment combined with low group homogeneity would be indicative of weaker polarization, and even no polarization in case of insignificant differences in attitude positions. Investigating the patterns of attitude positions, issue alignment, and group homogeneity jointly, and providing a solid characterization of attitude polarization, requires a novel framework that exploits the multidimensional nature of attitudes and overcomes the limitations of previous approaches that have strongly focused on attitude means.^40^

### The Attitudinal Space Framework

Natural scientists have developed multidimensional trait-based frameworks to better describe the functional diversity and structure of ecological communities.^41^^,^^42^^,^^43^ In functional ecology, the functional diversity and structure of an ecological community are evaluated through the measurement of functional traits.^44^ Species are then mapped onto a multidimensional space according to their trait characteristics. Several metrics derived from this multidimensional space, like functional specialization or dispersion, can describe community diversity and structure.^41^ Just as all aspects of functional diversity cannot be summarized in one metric, we contend that attitudinal diversity cannot be restricted to a single measure like mean attitude position.^45^^,^^46^ Hence, we propose that the attitudinal structure of a group of respondents can be defined as the distribution of individual respondents in an attitudinal space.^41^

**Figure 1:**
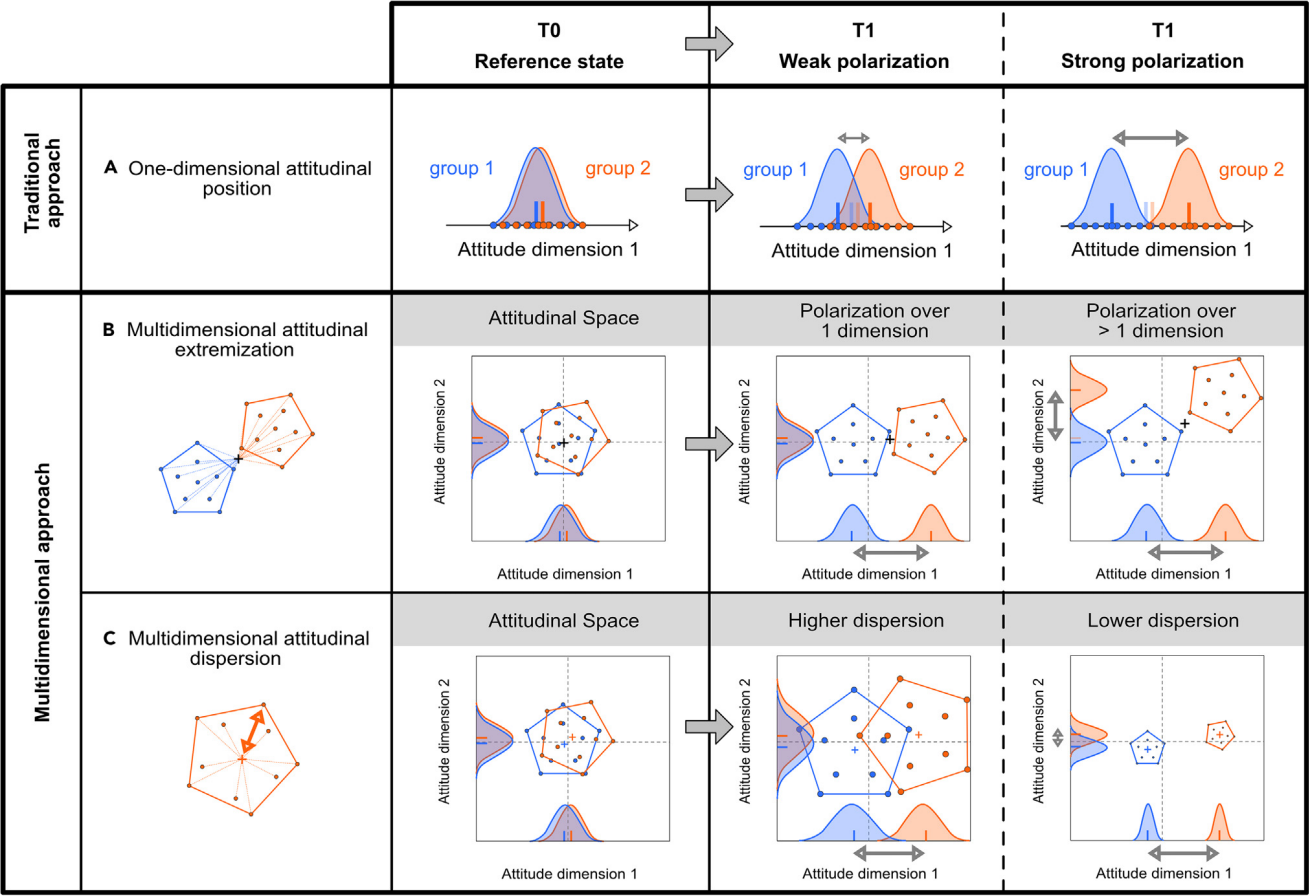
Traditional vs. novel multidimensional approaches to attitudinal polarization To date, assessments of attitudinal polarization have mostly used unidimensional approaches to evaluate the increasing difference between attitude positions of social groups over time (i.e., attitude averages at T0 and T1, blue and orange tick marks on X axes, Row A), neglecting the extent of attitudinal diversity. In contrast, the multidimensional nature of attitudes is accounted for in the attitudinal space framework (Rows B & C); for simplicity, only 2 dimensions are displayed. Attitudinal extremization (i.e., the average distance to the joint centroid of the entire population) quantifies group differences across multiple attitudinal dimensions and thus, issue alignment (Row B). Attitudinal dispersion (i.e., average distance of each individual to the centroid of its respective social group, Row C) assesses within-group homogeneity. Taken together, the attitudinal space framework allows to differentiate between weak and strong polarization.

We here introduce the ASF that, unlike traditional approaches using one-dimensional attitude positions, can quantify attitudinal diversity across multiple complementary attitudinal dimensions (Figure 1, Row A). Most importantly, the ASF helps to simultaneously address various polarization-related issues,^9^ including attitude alignment and group homogeneity, through the use of distance-based measures like multidimensional attitudinal extremization (Figure 1, Row B) and attitudinal dispersion (Figure 1, Row C), respectively. Attitudinal extremization quantifies the mean distance of individuals belonging to a specific group to the centroid of the entire population (i.e., across groups) in the attitudinal space. It therefore reflects attitude alignment as it measures the extent to which individuals of a group align their opinion across multiple attitudinal dimensions. As attitude polarization has recently been demonstrated to partly hinge on acrophily (i.e., the tendency to associate with others who have more extreme attitudes),^47^ this measure of attitudinal extremization is fundamental to better describe attitude polarization. Attitudinal dispersion measures the mean distance of individuals to the centroid of their own social group in the attitudinal space. It is therefore an indicator of group homogeneity that quantifies the clustering of attitudes within a single social group. Both measures are expressed as means over individual distances to centroids and range between 0 and 1, which allows for direct comparisons across individuals and social groups. The use of mean distances is consistent with both the literature on attitude polarization, which has largely focused on mean attitude positions, and the ecological literature, which has developed community-weighted means as indicators taking into account individual weights in the multidimensional space.

Together, these multidimensional measures provide a unique opportunity to quantitatively differentiate between weak and strong attitude polarization. Weak polarization is indicated by increasing attitude differences with low attitudinal extremization or high attitudinal dispersion (Figure 1, third column), while strong polarization is indicated by increasing attitude differences with high attitudinal extremization or low attitudinal dispersion (Figure 1, fourth column). The weakest case of attitude polarization involves both low extremization and high dispersion, while the strongest case of attitude polarization involves both high extremization and low dispersion.

The ASF is applicable to a broad range of social and social-ecological systems, affords cross-cultural comparisons, and accommodates different data types (continuous, ordinal, and categorical data) and missing data.^48^^,^^49^ We illustrate the relevance and the broad applicability of the ASF with two case studies. The first case study uses the American National Election Studies (ANES) dataset to investigate the phenomenon of affective polarization, whereby American partisans tend to increasingly dislike each other.^13^ In particular, we investigate whether this phenomenon entails increasing group homogeneity (i.e., decreased attitudinal dispersion in the attitudinal space). The second case study uses a dataset on attitudes toward wolf recolonization in Germany^50^ as an example of potential wildlife-related conflicts in Europe. We investigate attitude positions, issue alignment, and group homogeneity to test if polarization is stronger in regions with wolves compared to regions without wolves.

## Results

### Case study #1: Affective polarization between Democrat and Republican partisans

To investigate affective polarization between Democrat and Republican partisans, we selected 17 questions from the ANES dataset, related to feelings toward liberals vs. conservatives, toward Democratic vs. Republican party, toward Democratic vs. Republican vice-presidential and presidential candidates, as well as perception of specific traits of the presidential candidates (i.e., intelligent, knowledgeable, moral, leadership, care). Our measure of affective polarization to illustrate the use of the ASF therefore reflects a combined measure of partisan and elite dislike in the US electorate.^51^^,^^52^ We looked at affective polarization as a trend between 1988 and 2008 in each partisan group, in order to (i) test whether the ASF reveals increasing differences in attitude positions among Democrats and Republicans, (ii) test issue alignment in both partisan groups as measured by attitudinal extremization, and (iii) test group homogeneity as measured by attitudinal dispersion in each partisan group.

**Figure 2:**
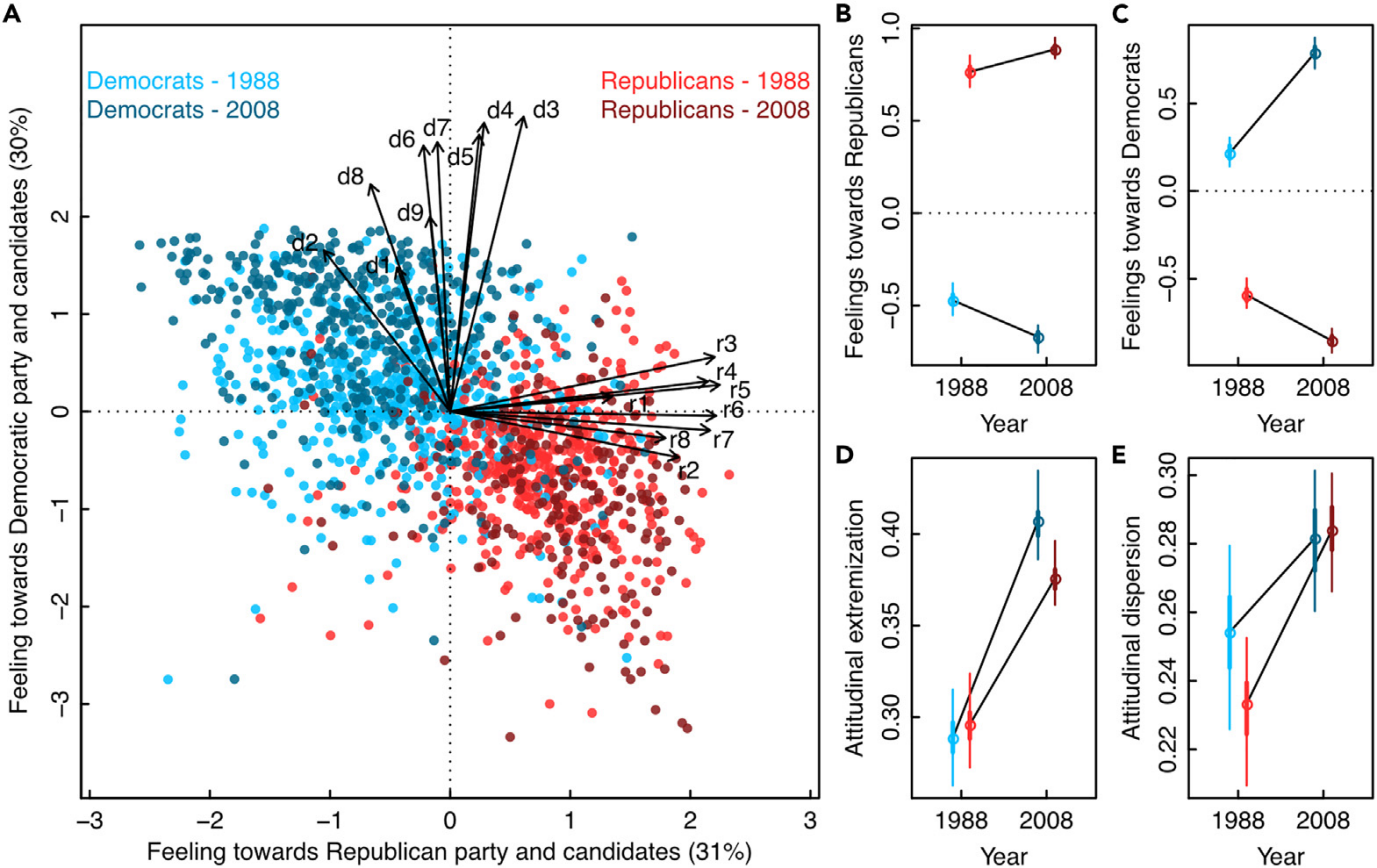
Increase in attitudinal dispersion in the American electorate The attitudinal space generated from the ANES dataset illustrates the position of partisans (strong Democrats and leaners in blue, strong Republicans and leaners in red) along two axes of feelings toward Republicans’ and Democrats’ party and candidates (Panel A; see Table S1 in supplemental information for details on questionnaire items d1-9 and r1-8), which explained 31% and 30% of the variance in the dataset, respectively. Traditional, one-dimensional approaches focusing on changes in mean attitude positions along Axis 1 (Panel B) and Axis 2 (Panel C) suggest an increase in affective polarization between the two groups from 1988 to 2008. The multidimensional attitudinal framework, however, reveals contrasting trends in different polarization measures, with a marked increase in attitudinal extremization (panel D) and in attitudinal dispersion (panel E). This together suggests an overall weaker phenomenon of affective polarization than previously assumed. All trends were significant (p-value < 0.05).

The two main dimensions of the resulting attitudinal space explained 61% of the variation in the dataset (see Methods). Consistent with previous findings, we found increasing affective polarization with a significant shift in mean attitude positions of Democrat and Republican partisans away from each other (Figure 2). This is illustrated by Republicans expressing increasingly positive feelings toward the Republican party and candidates, and increasingly negative feelings toward the Democratic party and candidates between 1988 and 2008, and vice versa for Republican partisans (Figures 2B and 2C). We also found a significant increase in attitudinal extremization in both partisan groups (+27.0% for Republicans, +40.8% for Democrats), reinforcing the trend of affective polarization along multiple attitude dimensions (Figure 2D). This demonstrates that each partisan group is increasingly driven by individuals occupying the extreme end of the attitudinal spectrum described in the attitudinal space and is therefore indicative of issue alignment. However, we also found a significant increase in attitudinal dispersion in both partisan groups (+21.9% for Republicans, +11.0% for Democrats), meaning that both groups are becoming increasingly heterogeneous through time. This is at odds with the hypothesis of party sorting, whereby partisan groups are expected to become increasingly homogeneous in their attitudes.^38^ Our results show the opposite pattern and therefore potentially hint at a slightly weaker affective polarization than anticipated, as the strongest polarization scenario would involve both higher extremization and lower dispersion. This is in line with the dual motivations theory suggesting an increased heterogeneity and ambivalence in partisans’ attitudes.^14^

### Case study #2: Polarization of attitudes toward wolves in Germany in regions with and without wolves

To investigate polarization of attitudes in a context of human-wildlife coexistence, we examined individual attitudes toward recolonizing wolves in regions with and without wolves in Germany, thus considering polarization as a state. Wolves returned to Germany in 2000 and since then have recolonized several regions in the Eastern and Northern parts of the country.^53^ The return of large carnivores in European landscapes is usually prone to heated debates and attitude polarization concerning their conservation and management.^54^^,^^55^ We were specifically interested in the broader public’s opinion because there has been a consistent narrative presenting the society as polarized on the issue of wolf return in Germany, driven by intense and often negative media coverage.^56^^,^^57^ We further focused on the distinction between regions with and without wolves as the distance to wolves and the inherent coexistence context are expected to impact attitudes.^50^^,^^58^ We used a phone survey conducted in 2017 targeting a representative sample of the German population, stratified by federal states.^50^ Here, we tested for (i) differences in attitude positions along two dimensions between regions with and without wolves, (ii) issue alignment, i.e., if regions with wolves had higher attitudinal extremization than regions without wolves, and (iii) regional homogeneity, i.e., if regions with wolves had lower attitudinal dispersion than regions without wolves, due to more concrete experience with human-wolf coexistence.

**Figure 3:**
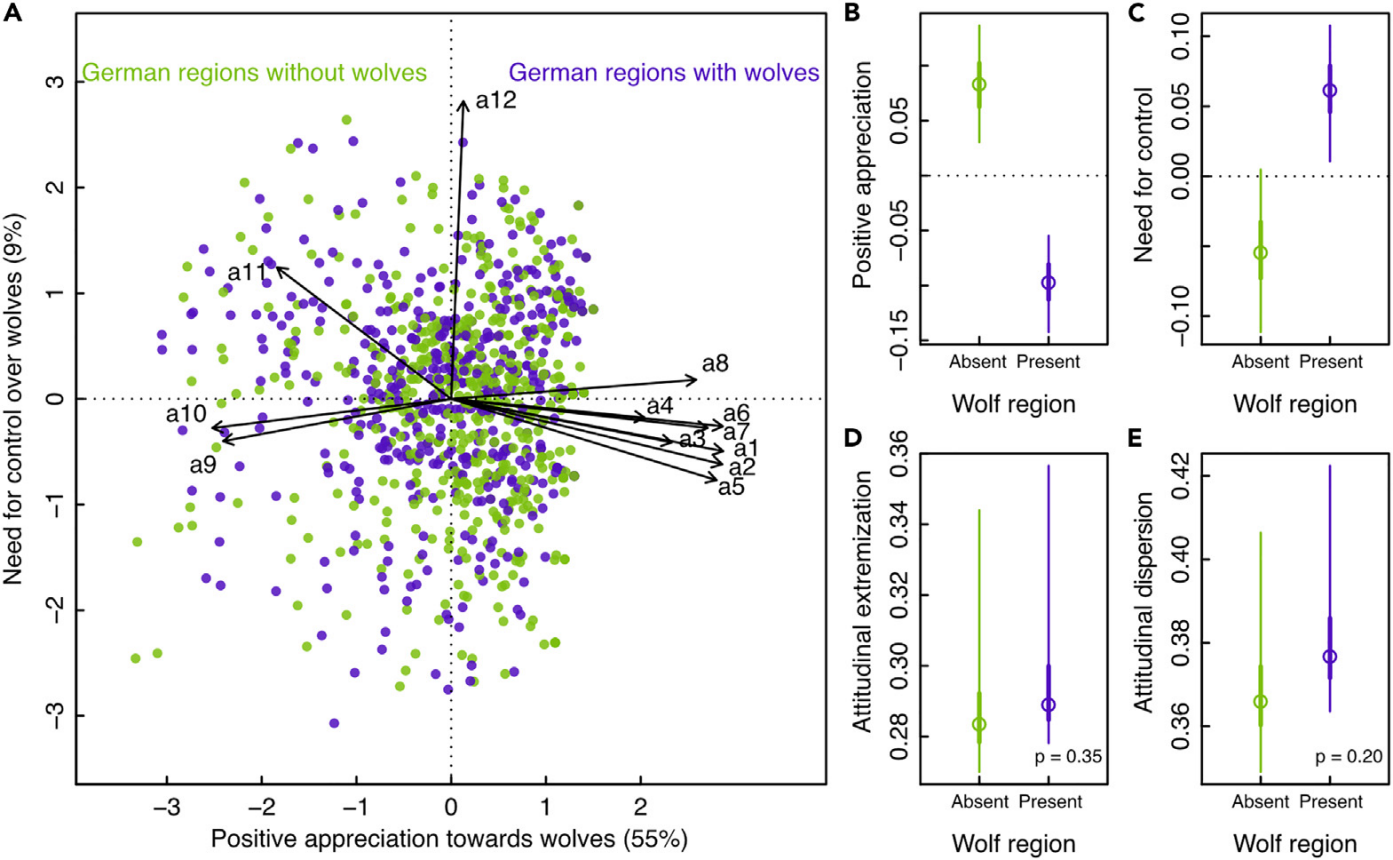
Weak polarization in attitudes toward wolves in Germany Attitudinal space based on survey respondents’ attitudes toward wolves in Germany, in regions without (green color) and with wolves (purple color) (Panel A; see Table S2 in supplemental information for details on questionnaire items a1-12). Attitudes significantly differed between regions with and without wolves (p-value < 0.05), with people in wolf regions expressing less positive appreciation (Panel B) and a greater desire to control wolf populations (Panel C). However, we found no significant differences in attitudinal extremization (Panel D) or dispersion (Panel E) between the two contexts, illustrating an overall weak polarization phenomenon. Numbers in brackets represent the percentage of variation explained by each attitudinal dimension.

The two dimensions of the resulting attitudinal space explained 64% of the variation in the dataset (Figure 3A). In concordance with a previous publication using this dataset,^50^ we found significant differences between regions with and without wolves in terms of mean attitude positions, with much greater appreciation of wolves in regions without wolves (Figure 3B) and a much greater desire for wolf population control in regions with wolves (Figure 3C). However, we did not detect any significant difference in attitudinal extremization or dispersion between regions with and without wolves (Figures 3D and 3E), showing similar levels of issue alignment and group homogeneity in the two regions. This means that human-wolf coexistence in Germany was not associated with strong attitude polarization in the broad public: the attitudinal space occupied by individuals living in regions with wolves was not characterized by more extreme or more homogeneous attitudes than the one occupied by individuals from regions without wolves. These findings suggest an overall weaker-than-expected polarization state solely characterized by differences in attitude positions, between regions with and without wolves.^50^

## Discussion

Our results illustrate the need to move beyond the traditional focus on attitude positions and to broaden research approaches to attitudinal diversity. The ASF offers a quantitative framework that jointly evaluates across- and within-group dynamics along multiple attitude dimensions. It therefore provides important new insights on the phenomenon of attitude polarization which is particularly relevant to sustainability science. In particular, the ASF allows joint assessments of issue alignment and group homogeneity, thereby differentiating between weak and strong attitudinal polarization, where weak polarization is indicated by increasing attitude differences with either low attitudinal extremization and/or high attitudinal dispersion and strong polarization is indicated by increasing attitude differences with either high attitudinal extremization and/or low attitudinal dispersion. Our two case studies demonstrate that the multidimensional nature of the ASF provides a better understanding of both the relative strength of affective polarization in the US electorate^13^ and public opinion polarization in regions recolonized by large carnivores in Europe.

Before discussing potential avenues for the ASF, we highlight three important limitations of this framework. First, the ASF is intended to better describe actual attitude polarization and does not encompass assessments of the causes of attitude polarization. Recent studies have started investigating the many drivers that can potentially influence attitudes, especially in political contexts.^21^ For instance, perceived polarization (i.e., how I think they think about us) is expected to play a role in reinforcing affective polarization.^19^^,^^21^ Similarly, mistrust in individuals who do not share one’s beliefs is expected to increase polarization,^34^ as is the spread of misinformation.^59^ Second, our framework does not account for the consequences of attitude polarization, like the multiple norm-, belief-, and behavioral-based decisions affected by polarization and influencing levels of cooperation, altruism, and social connections.^60^ For instance, one of the most debated aspects of polarization is the formation of echo chambers on social networks,^61^^,^^62^^,^^63^ whereby patterns of information sharing reinforce preexisting attitudes.^63^ However, although the ASF does not directly tap into drivers and consequences of polarization, it offers important and novel avenues for further explorations of the various aspects surrounding attitude polarization. This will be particularly helpful for designing efficient interventions to reduce attitude polarization and across-group animosity.^17^^,^^64^^,^^65^ Third, to illustrate the merits of the ASF, we chose to describe two case studies in which social groups were defined *a priori*. However, it has been shown that social groups can endogenously form over time over specific issues.^34^ The ASF can accommodate these considerations by using clustering approaches in the attitudinal space. The challenge then resides in carefully selecting the clustering algorithm to define a set of meaningful clusters representing the issue(s) at stake.^66^

The strength of the ASF lies in providing a broad conceptual frame that allows to quantitatively investigate the complexity of attitudinal diversity. Attitudinal diversity refers to the diversity of individual attitudes and the resulting complexity of the structure of social groups. The ASF embraces this complexity and offers complementary tools to visualize and analyze it. While, here, we focus on issue alignment (extremization) and group homogeneity (dispersion) as two potential diagnostic measures of attitude polarization, the ASF opens the door for applying a variety of distance-based attitude metrics and thus can address the complex mechanisms underpinning polarization (see Table 1 for a non-exhaustive list of potential metrics). For instance, among the multitude of factors potentially driving polarization,^9^ social networks can play a critical role in shaping individual attitudes. Indeed, individuals closer to each other in the multidimensional space are expected to communicate more frequently and efficiently, thus reinforcing polarization.^67^^,^^68^ In the ASF, another indicator called attitudinal originality is a measure of proximity with one’s closest neighbor in the attitudinal space^41^ and can be used to test the importance of individuals’ connectedness to peer group members in strengthening attitude polarization. This metric, combined with attitudinal extremization, can help tease apart the differential effects of homophily (i.e., the tendency to affiliate with others having similar views) and acrophily (i.e., the tendency to associate with others having more extreme views) on the emergence of attitude polarization.^47^ Further, attitudinal richness can be used to quantify the degree of overlap between two social groups in the attitudinal space. Such distance-based metrics inspired from functional ecology can be implemented in the ASF and be tailored to specific research questions related to attitudinal diversity^69^^,^^70^^,^^71^ (Table 1).

**Table 1:** Relevant metrics associated with the Attitudinal Space Framework

Metric	Description	Relevance
Attitude Position	Attitude position is the mean value of attitudinal traits along a specific attitudinal dimension, across all respondents of a social group.^41^	Attitude position has been the most widely used metric in attitude studies, to investigate attitudinal difference along a single dimension, between two or more social groups.
Attitudinal Extremization	Attitudinal extremization is the mean distance of respondents to the centroid of all respondents (i.e., across all social groups) in the attitudinal space.^83^^,^^84^	This measure represents the distinctiveness of attitudinal traits in the population and helps identify the social groups with the more extreme attitudes. Extremization provides critical information on social groups’ structure along multiple dimensions, hence on issue alignment.
Attitudinal Dispersion	Attitudinal dispersion is the mean distance of respondents to the centroid of their own social group. Dispersion is not influenced by extreme trait combinations like attitudinal richness.^48^	Attitudinal dispersion illustrates the distribution of the majority of respondents within a social group and therefore describes the extent to which this group is homogeneous in the sense that most respondents are clustered around the mean attitude of this group.
Attitudinal Richness	Attitudinal richness is the amount of attitudinal space filled by respondents in a social group^41^; it is calculated from the volume occupied by the convex hull defined by the most extreme points in a social group. Richness can be highly influenced by the sample size, and the TOP index^85^ may be most suitable for analysis of attitudinal richness.	In stakeholder analyses, attitudinal richness can be used to estimate the degree of attitudinal overlap between two, or more, social groups. In surveys targeting the broad public, it is critical that the sample is representative of the population, because of the influence of sample size on attitudinal richness.
Attitudinal Originality	Attitudinal originality is the mean distance between each respondent and its nearest neighbor in the attitudinal space.^84^	Attitudinal originality reflects the isolation of a respondent in the attitudinal space and measures the degree of uniqueness of individual attitudes in a social group.^84^ At the group level, it can identify groups that hold specific attitudes that cannot be found in other groups. At the individual level, it can help investigate the impact of one’s social network on individual attitudes.
Attitudinal Distance	Attitudinal distance is the distance between the centroids of two social groups in the attitudinal space.^86^	Attitudinal distance has been a central concept in studies of attitudinal polarization, along a single dimension. The attitudinal space offers the possibility to study distances between two, or more, social groups, along multiple attitude dimensions.
Attitudinal Entity	Attitudinal entities are unique combinations of attitudinal traits in the population of respondents.^87^	This measure can be used to avoid arbitrarily classifying respondents into social groups. The number of attitudinal entities in an attitudinal space can be compared to the potential number of attitudinal entities, i.e., the theoretical number of unique combinations based on the questionnaire items.
Attitudinal Evenness	Attitudinal evenness corresponds to the evenness of respondents’ distribution within a social group and is calculated using a minimum spanning tree that links all the points contained in the dimensional space with the minimum sum of branch lengths.^69^	Evenness is a promising metric to evaluate the regularity of distances between respondents of a specific social group and is in line with studies looking at the homogeneity of social groups in a multidimensional space.

Non-exhaustive list of distance-based attitude metrics inspired from functional ecology, relevant to investigate attitudinal diversity and mechanisms of attitude polarization.

The ASF illustrations presented in this article assumed that each individual respondent had an equivalent weight in its community, but where this is not the case, the framework can accommodate additional parameters describing the relative importance of individuals in the multidimensional space.^69^ For instance, sustainability studies on power imbalances and governance issues focusing on specific stakeholders can apply different weights to each individual in the attitudinal space by using social network analyses.^72^^,^^73^ These weights can then be applied to compute community-weighted means to account for the individual influences in the space,^43^ owing to, e.g., prestige, popularity, or social status. Such approaches can provide critical information in addition to the diagnostic measures we featured in our analyses. Referring back to the case study on the American electorate, partisan leaders who have a significant impact in their local social community (hence more weight in the attitudinal space) are expected to be clustered around the mean attitude position of the partisan group, thereby decreasing attitudinal dispersion and potentially strengthening attitude polarization overall.

Finally, we have presented the ASF as a promising tool to investigate attitudinal diversity, yet the strengths of this approach can be expanded to other contexts of quantitative social research. For instance, values, understood as guiding principles in one’s life, are critical components of cognitive processes underpinning people’s behavior,^74^^,^^75^ and understanding their dynamics of change is important to understand social and social-ecological trajectories of our societies,^76^^,^^77^ and identify transformative solutions. Designing a multidimensional value space and investigating value diversity in different social and social-ecological contexts with distance-based metrics would advance our understanding of value change. Similarly, as the diversity of emotions across cultures is gaining increasing research interest,^78^^,^^79^^,^^80^ we encourage future work on emotions to adapt this approach and define an emotional space to investigate emotional diversity. To conclude, the implementation of a multidimensional trait space from functional ecology to address issues in social sciences can provide innovative tools to better understand the dynamics of social-ecological systems.

## STAR★Methods

### Key resources table

**Table:** 

REAGENT or RESOURCE	SOURCE	IDENTIFIER
**Deposited data**		

ANES data	American National Election Studies (https://electionstudies.org/data-center/)	https://zenodo.org/record/7715985
German survey data	Arbieu et al.^50^	https://zenodo.org/record/7715985

**Software and algorithms**		

R Software (version 3.6.2)	R Core Team (2023). _R: A Language and Environment for Statistical Computing_. R Foundation for Statistical Computing, Vienna, Austria.	https://www.R-project.org/

### Resource availability

#### Lead contact

Further information and requests for resources and codes should be directed to the lead contact, Dr. Ugo Arbieu (ugo.arbieu@universite-paris-saclay.fr).

#### Materials availability

The data that support the findings of Case Study #1 study is publicly available on the website of the American National Election Studies (ANES dataset; https://electionstudies.org/data-center/). The data that supports the findings of Case Study #2 has been already published (Arbieu et al. 2019). Datasets and codes used in this study are deposited and publicly accessible on Zenodo (10.5281/zenodo.7715985).

### Experimental model and study participant details

#### Case study #1

We used the publicly available ANES dataset to investigate affective polarization in the American electorate. The ANES is a series of surveys approved by the American National Science Foundation going back to 1948. These surveys therefore address both adult males and adult females in the population and also contain information on ancestry, race or ethnicity, as they aim to be representative of the American electorate at the national level. Details on data collection can be found on the ANES webpage (https://electionstudies.org/data-center/).

#### Case study #2

We used a previously published dataset on attitudes toward wolves in Germany collected in 2017 to investigate polarization in a human-wildlife coexistence context.^50^ The social survey aimed to be representative of the German population, and included adult male and female respondents with an even sex ratio. This study did not collect information on ancestry, race or ethnicity. At the time of study (i.e., 2017), the Senckenberg Research Institute did not have an Institutional Review Board for studies involving human participants. The survey was designed in collaboration with social scientists from the Institute for Social-Ecological Research in Frankfurt am Main (ISOE, Germany), was voluntary, strictly anonymous and complied with European legislation. Details on data collection can be found in the Methods section of that article.

The effect of age, sex or ethnicity was beyond the scope of our study, and further studies could look into these effects on attitude polarization.

### Method details

#### Case study #1

We grouped respondents into Democrats and Republicans by pooling those who identified themselves as strong and weak democrats or republicans, respectively (item VCF0301). We selected 17 items that corresponded to feelings toward own party and presidential and vice-presidential candidates (see Table S1 in supplemental information for details on selected items). These items are in the form of feeling thermometers (ranging from 0 to 100, 4 items) and 4-points Liker scale items (13 items). These items were available for each presidential election between 1984 and 2008 and we chose to focus on the 20-year difference in attitudes between 1988 and 2008. We excluded respondents with >10% missing data from these 17 survey items, which resulted in a sample size of 1,602 individuals (541 and 406 Democrats in 1988 and 2008, respectively; 448 and 207 Republicans, respectively).

#### Case study #2

We grouped respondents in two categories, based on whether they lived in regions with or without wolf territories. We used 12 items of the original questionnaire (see Table S2 in supplemental information for details on selected items), which are in the form of 5-points Likert scale items (11 items) and one ordinal variable (1 item), and excluded respondents with >10% missing data from these 12 survey items, which resulted in a sample size of 946 individuals (440 in regions with wolves, 506 in regions without wolves).

### Quantification and statistical analysis

All analyses were done using R (version 3.6.2), and were similar in case study #1 and #2. First, we used a factor analysis with polychoric correlations and evaluated how many factors had an eigenvalue > 1^81^ and would therefore be needed to build the Attitudinal Space. In both case studies, two factors were deemed appropriate (see Figure S1 in the supplemental information). Second, because all items considered in this study were either numeric or ordinal, we used a Principal Component Analysis to build the Attitudinal Space. More details on the available options to build a robust multidimensional space can be found in recent publications.^42^ We used rotation for optimal interpretability of the principal components.^82^ In Case Study #1, we used a *promax* rotation as it accounts for the interfactor correlation between the two components in the multidimensional space (r = −0.55; CI = [-0.58;-0.52], see Table S3 in the supplemental information for factor loadings). In Case Study #2, we used a *varimax* rotation as the two principal components were not strongly correlated (r = −0.10; CI = [-0.16;-0.04], see Table S4 in the supplemental information for factor loadings). Third, we used the multidimFD function from Villéger et al.^69^ to calculate Attitudinal Diversity metrics for each social group, with a focus on Attitudinal Position, Extremization and Dispersion (see Table 1). Fourth, to obtain confidence intervals for each metric and each community, we used a bootstrap subsampling approach (1000 iterations): each metric was calculated with a random subsample of n = 150 individuals in each social group of Case Study #1 and n = 350 individuals in the two social groups of Case Study #2. Finally, we calculated p values to evaluate the difference between distributions of values for Attitudinal Positions, Extremization and Dispersion between 1988 and 2008 for each partisan group in Case Study #1, and between respondents from regions with and without wolves in Case Study #2.

## Data Availability

• Data: The datasets used in this article have been deposited at Zenodo: https://zenodo.org/record/7715985, and are publicly available as of the date of publication. The DOI is listed in the key resources table. • Code: The codes used for data analyses and data visualization have been deposited at Zenodo: https://zenodo.org/record/7715985, and are publicly available as of the date of publication. The DOI is listed in the key resources table. • Any additional information required to reanalyze the data reported in this paper is available from the lead contact upon request.
