# Supplementary material for: The attitudinal space framework: Embracing the multidimensionality of attitudinal diversity
Source: iScience. 2023 Jul 16;26(8):107340. doi: 10.1016/j.isci.2023.107340 (PMC10393727; doi:10.1016/j.isci.2023.107340)
Supplement: Document S1. Figures S1 and Tables S1–S4 [file mmc1.pdf]

iScience, Volume *26*

Supplemental information

The attitudinal space framework: Embracing the multidimensionality of attitudinal diversity

Ugo Arbieu, Jörg Albrecht, Katrin Böhning-Gaese, Lisa Lehnen, Matthias Schleuning, and Thomas Mueller

SUPPLEMENTAL INFORMATION

Table S1: Description of questionnaire items from the ANES survey used in Case Study #1

Table S2: Description of questionnaire items from the wolf survey used in Case Study #2

Figure S1: Eigen values from the Factor Analysis for both case studies

Table S3: Results of Principal Component Analysis in Case Study #1.

Table S4: Results of Principal Component Analysis in Case Study #2.

Table S1. Description of questionnaire items extracted from the American National Election Studies (ANES) dataset for years 1988 and 2008, and used to build the corresponding attitudinal space in the first case study to analyze affective polarization in the US electorate.

**Label**	**Description**	**Coding**
*Feeling thermometers to evaluate feelings about **some groups** in American society*		
d1	[VCF0211 in ANES dataset]<br>Feeling thermometer towards **Liberals**	From 0 to 100<br>0 = don't feel favorably toward the group<br>100 = feel favorably toward the group
r1	[VCF0212 in ANES dataset] Feeling thermometer towards **Conservatives**	From 0 to 100<br>0 = don't feel favorably toward the group<br>100 = feel favorably toward the group
d2	[VCF0218 in ANES dataset] Feeling thermometer towards the **Democratic Party**	From 0 to 100<br>0 = don't feel favorably toward the group<br>100 = feel favorably toward the group
r2	[VCF0224 in ANES dataset] Feeling thermometer towards the **Republican Party**	From 0 to 100<br>0 = don't feel favorably toward the group<br>100 = feel favorably toward the group
In your opinion, does the phrase [TRAIT] describe [POLITICAL FIGURE] extremely well, quite well, not too well or not well at all?		
d3	[VCF0350 in ANES dataset] POLITICAL FIGURE = Democratic Presidential candidate TRAIT = “intelligent”	From 1 to 4 [reverse coded]<br>1 = extremely well<br>2 = quite well<br>3 = not too well<br>4 = not well at all
d4	[VCF0354 in ANES dataset] POLITICAL FIGURE = Democratic Presidential candidate TRAIT = “knowledgeable”	From 1 to 4 [reverse coded]<br>1 = extremely well<br>2 = quite well<br>3 = not too well<br>4 = not well at all
d5	[VCF0355in ANES dataset] POLITICAL FIGURE = Democratic Presidential candidate TRAIT = “moral”	From 1 to 4 [reverse coded]<br>1 = extremely well<br>2 = quite well<br>3 = not too well<br>4 = not well at all
d6	[VCF0356 in ANES dataset] POLITICAL FIGURE = Democratic Presidential candidate TRAIT = “provides strong leadership”	From 1 to 4 [reverse coded]<br>1 = extremely well<br>2 = quite well<br>3 = not too well<br>4 = not well at all
d7	[VCF0357 in ANES dataset] POLITICAL FIGURE = Democratic Presidential candidate TRAIT = “really cares about people like you”	From 1 to 4 [reverse coded]<br>1 = extremely well<br>2 = quite well<br>3 = not too well<br>4 = not well at all
r3	[VCF0366 in ANES dataset] POLITICAL FIGURE = Republican Presidential candidate TRAIT = “knowledgeable”	From 1 to 4 [reverse coded]<br>1 = extremely well<br>2 = quite well<br>3 = not too well<br>4 = not well at all
r4	[VCF0367 in ANES dataset] POLITICAL FIGURE = Republican Presidential candidate TRAIT = “moral”	From 1 to 4 [reverse coded]<br>1 = extremely well<br>2 = quite well<br>3 = not too well<br>4 = not well at all
r5	[VCF0368 in ANES dataset] POLITICAL FIGURE = Republican Presidential candidate TRAIT = “provides strong leadership”	From 1 to 4 [reverse coded]<br>1 = extremely well<br>2 = quite well<br>3 = not too well

		4 = not well at all
r6	[VCF0369 in ANES dataset] POLITICAL FIGURE = Republican Presidential candidate TRAIT = “really cares about people like you”	From 1 to 4 [reverse coded]<br>1 = extremely well<br>2 = quite well<br>3 = not too well<br>4 = not well at all
Feeling thermometers to evaluate feelings about [POLITICAL FIGURE]		
d8	[VCF0424 in ANES dataset] Democratic Presidential Candidate	From 0 to 100<br>0 = don’t feel favorably toward the person and don’t care too much for that person<br>100 = feel favorably and warm toward the person)
d9	[VCF0425 in ANES dataset] Democratic Vice-Presidential Candidate	From 0 to 100<br>0 = don’t feel favorably toward the person and don’t care too much for that person<br>100 = feel favorably and warm toward the person)
r7	[VCF0426 in ANES dataset] Republican Presidential Candidate	From 0 to 100<br>0 = don’t feel favorably toward the person and don’t care too much for that person<br>100 = feel favorably and warm toward the person)
r8	[VCF0427 in ANES dataset] Republican Vice-Presidential Candidate	From 0 to 100<br>0 = don’t feel favorably toward the person and don’t care too much for that person<br>100 = feel favorably and warm toward the person)

Table S2. Description of questionnaire items extracted from Arbieu et al. (2019) used to build the corresponding attitudinal space in the second case study on attitudes towards wolves in Germany.

Label	Description	Coding
a1	What is your opinion about wild wolves?	From 1 to 5 [reverse coded]<br>1 = very positive<br>2 = positive<br>3 = neutral<br>4 = negative<br>5 = very negative
a2	How do you feel about wild wolves living in Germany again?	From 1 to 5 [reverse coded]<br>1 = very positive<br>2 = positive<br>3 = neutral<br>4 = negative<br>5 = very negative
a3	Would you enjoy seeing wild wolves or wild wolves' signs (e.g. tracks, hair, howls…) in the wild in Germany?	From 1 to 5 [reverse coded]<br>1 = would enjoy very much<br>2 = would enjoy<br>3 = neutral<br>4 = would not enjoy<br>5 = would not enjoy at all
a4	How far from your place of residence would you tolerate the presence of wild wolves (in km)?	Continuous variable transformed into ordinal variable<br>1 = < 1km<br>2 = 1 – 10km<br>3 = > 10km and ≤ 100km<br>4 = > 100km
a5	What evolution in the wild wolf population of Germany would you wish for the future?	From 1 to 4 [reverse coded]<br>1 = more wolves<br>2 = as many wolves as today<br>3 = less wolves<br>4 = no wolves at all
To what extent do you agree with the following statements?		
a6	Wild wolves have, like other animals, a right to live in Germany.	From 1 to 5 [reverse coded]<br>1 = strongly agree<br>2 = agree<br>3 = neutral<br>4 = disagree<br>5 = strongly disagree
a7	Wild wolves should only live in Nature reserves and other Protected Areas	From 1 to 5 [reverse coded]<br>1 = strongly agree<br>2 = agree<br>3 = neutral<br>4 = disagree<br>5 = strongly disagree
a8	The presence of wild wolves increases the value of a landscape, whether I get to see them or not	From 1 to 5 [reverse coded]<br>1 = strongly agree<br>2 = agree<br>3 = neutral<br>4 = disagree<br>5 = strongly disagree
a9	Because of the presence of wild wolves, I would be scared to walk alone in the forest	From 1 to 5 [reverse coded]<br>1 = strongly agree<br>2 = agree<br>3 = neutral<br>4 = disagree<br>5 = strongly disagree

a10	The presence of wild wolves would negatively affect my leisure activities	From 1 to 5 [reverse coded]<br>1 = strongly agree<br>2 = agree<br>3 = neutral<br>4 = disagree<br>5 = strongly disagree
a11	The number of wolves should be controlled by human shooting	From 1 to 5 [reverse coded]<br>1 = strongly agree<br>2 = agree<br>3 = neutral<br>4 = disagree<br>5 = strongly disagree
a12	Only those wolves who cause problems and damages should be controlled in Germany through scaring, capturing, relocating or shooting	From 1 to 5 [reverse coded]<br>1 = strongly agree<br>2 = agree<br>3 = neutral<br>4 = disagree<br>5 = strongly disagree

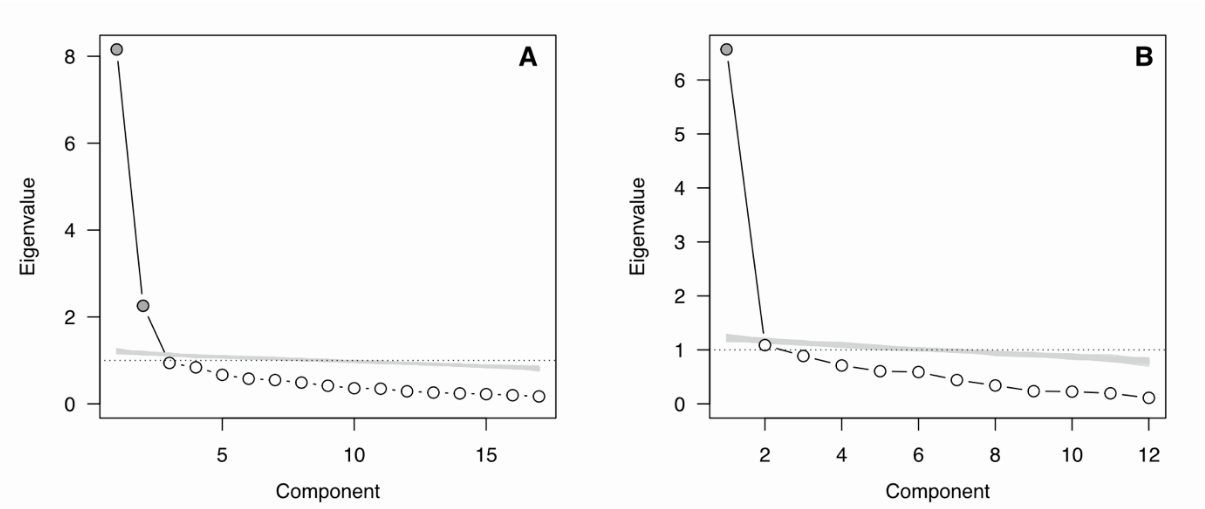

Figure S1. Eigen values from the Factor Analysis performed in (A) Case Study #1 and (B) Case Study #2. In both cases, we retained factors that had an eigen value > 1 (Kaiser rule) and therefore chose two principal components. The grey lines represent simulated eigen values obtained from random permutations in the respective datasets.

Table S3. Details of factors loadings and explained variance of the principal component analysis in Case Study #1.

Questionnaire items	Principal Component 1	Principal Component 2
d1	-0.17	**0.44**
r1	**0.52**	0.05
d2	**-0.41**	**0.50**
r2	**0.73**	-0.14
d3	0.24	**0.91**
d4	0.11	**0.89**
d5	0.09	**0.85**
d6	-0.09	**0.82**
d7	-0.04	**0.83**
r3	**0.85**	0.17
r4	**0.83**	0.09
r5	**0.87**	0.08
r6	**0.86**	-0.01
d8	-0.26	**0.70**
d9	-0.06	**0.60**
r7	**0.84**	-0.06
r8	**0.69**	-0.08
Proportion of variance explained	30.8 %	29.7 %
Cumulative variance explained	30.8 %	60.5 %

Table S4. Details of factors loadings and explained variance of the principal component analysis in Case Study #1

Questionnaire items	Principal Component 1	Principal Component 2
a1	**0.86**	-0.16
a2	**0.86**	-0.20
a3	**0.70**	-0.13
a4	**0.61**	-0.06
a5	**0.84**	-0.25
a6	**0.86**	-0.08
a7	**0.81**	-0.09
a8	**0.78**	0.06
a9	**-0.72**	-0.13
a10	**-0.76**	-0.09
a11	**-0.55**	**0.41**
a12	0.04	**0.92**
Proportion of variance explained	53.8 %	10.0 %
Cumulative variance explained	53.8 %	63.8 %
